# Association Between Dietary Habits and Their Scores With the 9.9‐Year Incidence of Dyslipidemia in an Urban Adult Population: Findings From a Prospective Cohort Study

**DOI:** 10.1002/fsn3.72249

**Published:** 2026-08-13

**Authors:** Mohammadtaghi Sarebanhassanabadi, Abolfazl Mohammadi, Pedro Marques‐Vidal, Seyedmostafa Seyedhosseini, Seyedeh Mahdieh Namayandeh, Hanieh Montazeri Hedesh, Sima Mozafari

**Affiliations:** ^1^ Yazd Cardiovascular Research Center, Non‐Communicable Diseases Research Institute Shahid Sadoughi University of Medical Sciences Yazd Iran; ^2^ Department of Medicine, Internal Medicine Lausanne University Hospital, University of Lausanne Lausanne Switzerland; ^3^ Clinical Research Development Center, Afshar Hospital Shahid Sadoughi University of Medical Sciences Yazd Iran

**Keywords:** dietary habits, dyslipidemia, incidence

## Abstract

Dyslipidemia elevates the risk of metabolic disorders and cardiovascular disease (CVD). While various dietary and lifestyle factors contribute to dyslipidemia, healthier eating habits may decrease this risk. This longitudinal study investigated the association between dietary habits score (DHS) and the 9.9‐year risk of dyslipidemia among urban adults. A total of 1042 adults without dyslipidemia at baseline were enrolled, and 693 participants who completed follow‐up were included in the final analysis. Dietary habits were assessed in 2005–2006, and participants were followed for an average of 9.9 years. DHS was calculated for each participant by summing the hazard ratios (HRs) associated with individual dietary habits in relation to dyslipidemia risk, thereby generating a cumulative score reflecting overall dietary habits quality. A higher DHS indicates unhealthier dietary habits. The association between DHS and the incidence of dyslipidemia was analyzed using Cox regression. During the 9.9‐year follow‐up, the incidence of dyslipidemia was 65.8%. After adjustment for confounders, the incidence of dyslipidemia (HR; 95% CI) was found to be associated with poor weight management (HR = 1.68; 95% CI: 1.14–2.47), low salad consumption (HR = 2.46; 95% CI: 1.56–3.90), and frequent fast‐food intake (HR = 1.90; 95% CI: 1.08–3.34). Overall, individuals in the highest DHS quartile exhibited a greater risk of dyslipidemia (HR = 2.58; 95% CI: 1.50–4.43, *p* = 0.001) compared with the lowest quartile of DHS. The same positive association was found in males (HR = 3.56; 95% CI: 1.95–6.51, *p* = 0.034). The findings indicate a significant positive association between poor weight management, low salad consumption, frequent fast‐food intake, and higher DHS with the 9.9‐year incidence of dyslipidemia. Further large‐scale prospective studies are needed to validate these findings.

## Graphical abstract

Dyslipidemia is a major risk factor for cardiometabolic diseases, and overall dietary habits may better predict its long‐term risk than individual eating habits. In this 9.9‐year prospective cohort study, low salad intake, frequent fast‐food intake, and poor weight management were independently associated with a higher risk of incident dyslipidemia. A higher dietary habits score (DHS), representing the combined influence of these habits, was also associated with a significantly increased long‐term risk. DHS may serve as a simple tool for identifying individuals at high risk of dyslipidemia and guiding targeted dietary interventions.

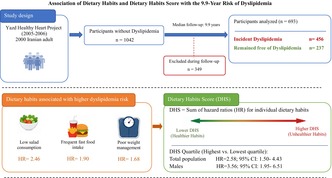

**Abbreviations**
BMI: body mass index
CI: confidence interval
CVD: cardiovascular disease
DBP: diastolic blood pressure
DHS: dietary habits score
DQI‐I: dietary quality index international
FBS: fasting blood sugar
HDL‐C: high‐density lipoprotein cholesterol
HEI‐2015: Healthy Eating Index‐2015
HR: hazard ratio
IPAQ: International Physical Activity Questionnaire
LDL‐C: low‐density lipoprotein cholesterol
SBP: systolic blood pressure
SD: standard deviation
STROBE: strengthening the reporting of observational studies in epidemiology
TC: total cholesterol
TG: triglyceride
WC: waist circumference
WHO: World Health Organization
YHHP: Yazd Healthy Heart Project

## 1 Introduction

Dyslipidemia is a metabolic disorder characterized by increased levels of total cholesterol (TC), low‐density lipoprotein cholesterol (LDL‐C), and triglyceride (TG), as well as diminished levels of high‐density lipoprotein cholesterol (HDL‐C) (Berberich and Hegele 2022; Lin et al. 2018). It has become a major public health issue in many regions, primarily because of its high prevalence and its links to serious health conditions (Berberich and Hegele 2022; Lin et al. 2018; Pirillo et al. 2021; Lee et al. 2017; Kargar and Ansari 2023). A study revealed that 54% of individuals in the Middle East are affected by dyslipidemia (Kargar and Ansari 2023). People with dyslipidemia have at least a two‐fold higher risk of developing cardiovascular disease (CVD) compared with those with normal lipid levels (Mozaffarian et al. 2016). On the other hand, individuals with dyslipidemia (high TG levels and low HDL‐C) are more susceptible to CVD, especially if they are also diagnosed with diabetes (Lee et al. 2017). Therefore, it is critical to identify and address the primary causes and contributing factors of dyslipidemia to achieve effective management and prevention.

The etiology of dyslipidemia is multifactorial, including genetic factors, lifestyle choices, and environmental influences, with dietary habits being a key aspect (Li et al. 2023; Alam et al. 2024). Research has established the impact of specific dietary elements—including intake of fruits, vegetables, fats, and salt, as well as cooking techniques—on lipid profiles (Agarwala et al. 2022).

Dietary habits, including the consumption of processed meats, high‐fat dairy products, and excessive salt, have been linked to unfavorable lipid profiles, whereas diets rich in fruits, vegetables, and whole grains are associated with improved lipid profiles (Wang et al. 2023; Yokoyama et al. 2017). However, because dietary habits are complex and foods are consumed together, isolating the effect of any single component is difficult. To better understand the impact of these choices on dyslipidemia, it is crucial to develop comprehensive dietary indices. While general dietary indices are valuable, they may not adequately reflect the culturally specific dietary patterns of urban Iranian adults. To address this, we constructed a population‐specific dietary habit score (DHS) for this cohort, where individual habits were weighted by their observed association with dyslipidemia risk. This approach provides a relevant metric to investigate the long‐term impact of cumulative dietary habits on dyslipidemia risk. This approach enables more effective dietary recommendations, ultimately promoting better health (Sarebanhassanabadi et al. 2020). A range of assessment scales is utilized to measure different factors, including dietary habits, by utilizing a series of focused questions aimed at identifying distinct attributes (Sarebanhassanabadi et al. 2020; Nouri, Gerami, et al. 2023). The overall score, calculated by summing all weighted questions and adjusting for potential confounders, represents specific individual attributes. Consequently, the establishment of a robust scoring system is essential for accurately estimating the risk of clinical outcomes related to specific behaviors. However, cohort evidence on the association between a DHS and the long‐term incidence of dyslipidemia, particularly in specific populations like urban Iranian adults, remains limited. Consequently, we aimed to investigate this association within the framework of the Yazd Healthy Heart Project (YHHP). This study examines the impact of overall dietary habits, summarized by a DHS, on the development of dyslipidemia over nearly a decade of follow‐up. We hypothesize that a higher DHS is associated with a higher incidence of dyslipidemia.

## 2 Materials and Methods

### 2.1 Study Population

**FIGURE 1:**
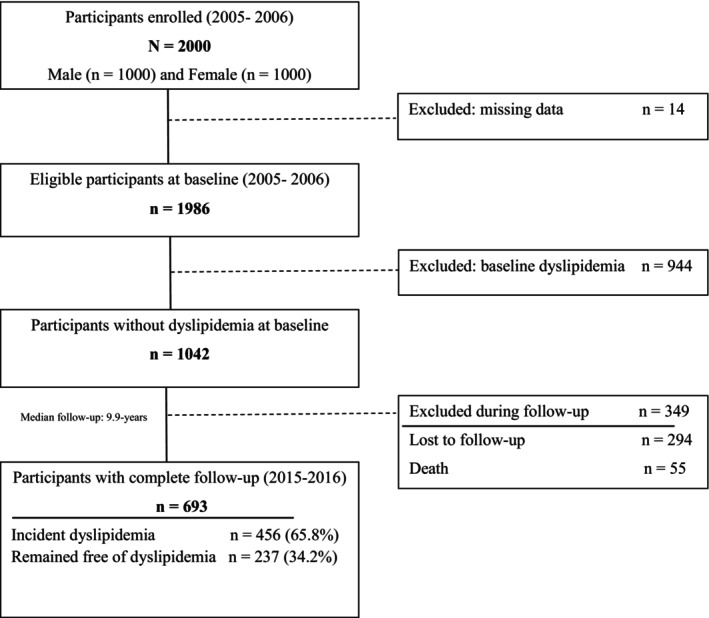
Flowchart of participant enrollment and follow‐up in the Yazd Healthy Heart Project, Yazd, Iran.

This study was conducted based on data from the YHHP in Yazd, Iran. In the first phase, 2000 people (1000 males and 1000 females) aged between 20 and 74 years were selected using a random sampling method during 2005–2006, and after 9.9‐year follow‐up, eligible participants were invited for the second phase. A detailed flow of participant inclusion is provided in Figure 1. Briefly, of the 2000 initially recruited individuals, 14 were excluded due to missing data. From the remaining 1986 participants, 944 with prevalent dyslipidemia at baseline were excluded from the incidence analysis. The final analysis included 693 participants without dyslipidemia at baseline who completed the 9.9‐year follow‐up.

This study undertook a comprehensive investigation into the effect of factors on increasing the risk of dyslipidemia. Information related to the demographics, biochemical factors (fasting blood sugar (FBS), TC, TG, LDL‐C, and HDL‐C levels), anthropometry (weight, height, and waist circumference (WC)), lifestyle (dietary habits, physical activity, and smoking status), and other covariates (economic status and education levels) was recorded. Participants with dyslipidemia at the baseline were excluded from the study.

The survey was approved by the Ethics Committee at the Shahid Sadoughi University of Medical Sciences Ethics Committee (Ethics Code: IR.SSU.MEDICINE.REC.1395.287) and conducted in accordance with the Strengthening the Reporting of Observational Studies in Epidemiology (STROBE) guideline. Every participant provided informed consent for using their data for analysis at the initial and the follow‐up phase.

### 2.2 Assessment of Dietary Habits Score (DHS)

The DHS is a weighted sum of 14 binary dietary habits (controlling body weight, eating salad, frequent fast‐food consumption, adding salt at the table, using measuring cups for salt or oil, removing poultry skin, dining out, consuming high‐fat dairy, removing visible meat fat, using mayonnaise, cooking methods (frying, roasting, boiling), and checking food labels). For each habit: healthy behavior receives a score of 1, and unhealthy behavior receives a score equal to the hazard ratio (HR) reported in Tables 2 and 3 of this manuscript (total population in Table 2; sex‐specific in Table 3). The formula is: DHS = Σ (score of habit₁ + habit_2_ + … + habit₁₄), where the score is either 1 (healthy) or the corresponding HR (unhealthy). A higher DHS means unhealthier overall dietary habits. Calculation steps: collect dietary habits at baseline via interview, assign 1 or the HR to each answer, sum all 14 scores to obtain raw DHS, and then divide participants into quartiles (Q1 to Q4). Tables 4 and 5 show the quartile cut‐offs and the graded increase in dyslipidemia risk from Q1 (lowest DHS) to Q4 (highest DHS).

### 2.3 Assessment of Dyslipidemia

Individuals with one or more of the following conditions are classified as having dyslipidemia: LDL‐C levels ≥ 160 mg/dL, TG levels > 200 mg/dL, TC levels ≥ 240 mg/dL, HDL‐C levels < 40 mg/dL, or presence of hypolipidemic medication (Lin et al. 2018). Five ml of blood obtained after overnight fasting was collected in the morning. Bioassays were conducted in a single laboratory, and all analyses of LDL‐C and HDL‐C were assessed using Bionic kits (Bionic Company, Tehran, Iran), while TG were assessed using a diagnostic kit (Pars Azmoon Inc., Tehran, Iran).

### 2.4 Assessment of Variables

Firstly, confounding variables (age, sex, education level, economic status, physical activity, and smoking) were assessed via interviewer‐administered questionnaires. To provide more details, the education level was categorized into three groups based on the highest attained level: primary (elementary or guidance school), high school (diploma), and academic (university degree). The economic status was a composite score based on three indicators: reported household income, ownership of a private car, and home area (in square meters). A score was assigned to each indicator, and the sum was calculated for each participant. Participants were then classified into low, moderate, and high economic status groups based on tertiles of the total score. Physical activity was assessed using the International Physical Activity Questionnaire (IPAQ). Participants were categorized into three levels based on their estimated weekly energy expenditure: low (< 600 kcal/week), moderate (600–1200 kcal/week), and vigorous (> 1200 kcal/week). Additionally, anthropometric measurements (weight, height, and WC) were taken using standardized equipment and techniques, and the body mass index (BMI) was calculated. Blood pressure was recorded twice using a digital monitor (M6 Comfort from Omron Co., Osaka, Japan) after 5 min of rest, and the subsequent measurements were taken at 5‐min intervals. In the next step, analyses were conducted using the average systolic blood pressure (SBP) and diastolic blood pressure (DBP) measurements. Also, we assessed the FBS in the laboratory by a biochemical autoanalyzer (BT 3000, Italy) and a diagnostics kit (Pars Azmoon Inc., Tehran, Iran).

### 2.5 Statistical Analysis

Data were presented as mean ± standard deviation (SD), unless otherwise specified. Categorical variables across DHS quartiles were compared using chi‐squared tests, while quantitative variables were analyzed using ANOVA. For continuous variables, an independent samples *t*‐test was used to compare the two groups.

The association between dietary habits and DHS with the 9.9‐year incidence of dyslipidemia was assessed using the Cox proportional hazards ratio in crude, Model I, and Model II analyses. Model I adjusted for age and sex (when analyzing the total population), whereas Model II included further adjustments for variables such as physical activity, smoking behavior, economic status, and educational levels. Analyses were conducted separately for males, females, and the overall population using both unadjusted and multivariable‐adjusted models. All statistical analyses were conducted in SPSS version 19 (IBM Corporation, New York, USA); significance was defined as *p* < 0.05.

## 3 Results

### 3.1 Population and Participant Follow‐Up

The cohort study began with 2000 participants, consisting of 1000 males and 1000 females, during 2005–2006. Follow‐up measurements were conducted during 2015–2016 to assess the association between dietary habits and their scores, as well as the 9.9‐year incidence of dyslipidemia in an urban adult population. In the initial phase of this study, 14 participants were excluded from the analysis because of missing data (Figure 1). The analysis revealed that 944 participants (47.5%) had dyslipidemia at the study baseline, while 1042 participants (52.5%) did not. In the second phase, 349 participants (33.5%) did not complete the follow‐up, and only 693 participants (66.5%) were included in the final analysis for dyslipidemia (Figure 1). The results indicated that the incidence of dyslipidemia in the adult population was 65.8%, while 34.2% of the participants did not have dyslipidemia.

### 3.2 Baseline Characteristics of Participants

We compared the baseline characteristics of participants who completed the follow‐up (*N* = 693) with those who did not complete the follow‐up (*N* = 349). The analysis showed that the groups differed significantly in age, sex, weight, WC, economic status, and education level. However, no significant differences were found in key clinical and metabolic variables, including lipid profiles, blood pressure, FBS, smoking, and physical activity.

In addition, Table 1 presents the baseline characteristics of the study population, highlighting the differences between those who developed dyslipidemia and those who did not. Significant differences were observed between the two groups in terms of age, weight, BMI, SBP, DBP, FBS, TG, TC, LDL‐C, HDL‐C, WC, levels of physical activity, and educational attainment (*p* < 0.05).

**TABLE 1:** Baseline characteristics of study population between participants who developed dyslipidemia and subjects without dyslipidemia.

Variable	Total (*n* = 1986)	Non‐ dyslipidemia (*n* = 1042)	Dyslipidemia (*n* = 944)	*p* value
Age (years)	48.7 ± 15.2	46.0 ± 15.9	51.6 ± 13.8	0.001
Male sex (%)	990 (49.8)	535 (51.3)	455 (48.2)	0.164
Educational level (%)				0.001
Primary	1177 (59.3)	551 (54.5)	626 (67.5)	
High school	583 (29.4)	362 (35.8)	221 (23.8)	
Academic	178 (9.1)	98 (9.7)	80 (8.6)	
Economic status (%)				0.82
Low	273 (31.0)	141 (30.1)	132 (32.1)	
Moderate	351 (39.8)	189 (40.3)	162 (39.3)	
High	257 (29.2)	139 (29.6)	118 (28.6)	
Smoking status (yes, %)	348 (17.5)	182 (17.5)	166 (17.6)	0.787
Physical activity (%)				0.011
Low	931 (68.6)	454 (64.9)	477 (72.4)	
Moderate	364 (26.8)	211 (30.2)	153 (23.2)	
High	63 (4.6)	34 (4.9)	29 (4.4)	
Anthropometry				
Weight (Kg)	70.8 ± 12.9	69.1 ± 12.8	72.6 ± 12.8	0.001
Body mass index (Kg/m^2^)	26.1 ± 4.48	25.4 ± 4.5	26.9 ± 4.2	0.001
Waist circumference (cm)	93.5 ± 12.2	90.7 ± 12.4	96.5 ± 11.2	0.001
Blood pressure (mmHg)				
Systolic	128 ± 16	125 ± 15	131 ± 16	0.001
Diastolic	83 ± 9	81 ± 8	84 ± 9	0.001
Fast blood sugar (mg/dL)	102.8 ± 45.6	93.7 ± 32.1	112.8 ± 55.1	0.001
Lipid levels (mg/dL)				
Triglyceride	175.4 ± 107.9	115.7 ± 40.3	241.3 ± 120.1	0.001
Total cholesterol	199.1 ± 59.4	179.7 ± 31.7	220.4 ± 73.8	0.001
LDL‐cholesterol	108.5 ± 36.4	98.7 ± 28.6	119.9 ± 40.9	0.001
HDL‐cholesterol	53.9 ± 13.7	57.4 ± 11.1	50 ± 15.1	0.001

*Note:* Results are expressed as number of participants (column percentage) for categorical variables and as mean ± standard deviation for continuous variables. Between groups comparisons performed using chi‐squared for categorical variables and student's *t*‐test for continuous variables.

Table 2 illustrates the association between dietary habits and the 9.9‐year incidence of dyslipidemia in the total population. After adjusting for all potential confounders, participants who did not have a weight management program (HR = 1.68; 95% CI: 1.14–2.47), those who did not consume salad (HR = 2.46; 95% CI: 1.56–3.90), and participants who frequently ate fast food (HR = 1.90; 95% CI: 1.08–3.34) were at increased risk after a 9.9‐year follow‐up.

**TABLE 2:** Hazard ratio (95% confidence interval) for dyslipidemia development based on dietary habits in the total population of the Yazd Healthy Heart Project, Yazd, Iran.

	Crude	Adjusted (Model I)	Adjusted (Model II)
Eating behaviors			
Weight control program (no)	1.20 (0.98–1.46)	1.18 (0.97–1.45)	**1.68 (1.14–2.47)**
Attention to food labels (no)	1.05 (0.87–1.28)	1.01 (0.82–1.23)	**0.52 (0.33–0.83)**
Dining out (yes)	0.98 (0.80–1.20)	1.03 (0.83–1.27)	1.00 (0.69–1.44)
Dietary behaviors			
Salt intake with food when eating (yes)	0.85 (0.69–1.04)	0.88 (0.71–1.09)	0.94 (0.61–1.44)
Special measuring cup for adding salt (no)	1.39 (1.13–1.71)	1.41 (1.15–1.74)	1.23 (0.80–1.90)
Remove of poultry skin (no)	0.60 (0.41–0.87)	0.58 (0.40–0.86)	**0.39 (0.21–0.89)**
High‐fat dairy (yes)	0.93 (0.79–1.24)	0.99 (0.80–1.24)	0.88 (0.58–1.32)
High‐fat meat (yes)	1.21 (0.97–1.53)	1.25 (0.99–1.57)	1.30 (0.79–2.13)
Fried food (yes)	0.78 (0.64–0.95)	0.79 (0.64–0.97)	0.77 (0.52–1.15)
Consuming mayonnaise (yes)	0.97 (0.77–1.22)	1.01 (0.81–1.26)	0.99 (0.65–1.52)
Special measuring cup for adding oil (no)	1.17 (0.96–1.43)	1.21 (0.99–1.48)	0.88 (0.57–1.34)
Fast food (yes)	1.00 (0.77–1.28)	1.10 (0.82–1.47)	**1.90 (1.08–3.34)**
Eating salad (no)	1.38 (1.11–1.72)	1.36 (1.09–1.71)	**2.46 (1.56–3.90)**
Boiled food (no)	0.82 (0.68–1.10)	0.85 (0.69–1.05)	0.84 (0.56–1.25)

*Note:* Results are expressed as hazard ratios and (95% confidence interval). Model I: Adjusted for age and sex; Model II: Adjusted for age, sex, smoking, economic status, physical activity and educational level. Significant (*p* < 0.05) results are indicated in bold.

The association between dietary habits and the incidence of dyslipidemia in both males and females approximately over 10 years is summarized in Table 3. The analysis revealed distinct gender‐specific associations with dyslipidemia risk. Among males, increased risk was associated with the absence of salad consumption, nonparticipation in a weight control program, and the consumption of high‐fat meat after adjusting for confounders. In contrast, among females, a significant association was observed only for fast food consumption in the adjusted model, with no other factors demonstrating a significant relationship with dyslipidemia risk.

**TABLE 3:** Hazard ratio (95% confidence interval) for dyslipidemia based on dietary habits, stratified by gender, the Yazd Healthy Heart Project, Yazd, Iran.

	Male			Female		
	Crude	Adjusted (Model I)	Adjusted (Model II)	Crude	Adjusted (Model I)	Adjusted (Model II)
Eating behaviors						
Weight control program (no)	1.34 (1.03–1.75)	1.35 (1.03–1.76)	**1.82 (1.15–2.87)**	1.04 (0.77–1.41)	0.95 (0.70–1.29)	1.42 (0.63–3.19)
Attention to food label (no)	0.91 (0.70–1.17)	0.91 (0.70–1.19)	1.02 (0.64–1.63)	1.26 (0.94–1.70)	1.10 (0.81–1.51)	1.47 (0.58–3.72)
Dining out (yes)	0.68 (0.49–0.94)	0.65 (0.47–0.91)	0.82 (0.48–1.40)	0.76 (0.50–1.14)	0.88 (0.57–1.35)	1.32 (0.46–3.70)
Dietary behaviors						
Salt intake with food when eating (yes)	0.87 (0.67–1.13)	0.83 (0.63–1.11)	0.96 (0.58–1.58)	0.80 (0.57–1.10)	0.88 (0.62–1.23)	0.81 (0.34–1.95)
Using special measuring cup for adding salt (no)	1.55 (1.17–2.04)	1.56 (1.18–2.06)	1.32 (0.80–2.18)	1.19 (0.86–1.65)	1.17 (0.85–1.62)	1.61 (0.59–4.37)
Removing of poultry skin (no)	0.55 (0.35–0.86)	0.55 (0.35–0.86)	0.48 (0.22–1.03)	0.77 (0.38–1.58)	0.75 (0.37–1.53)	1.19 (0.15–9.70)
High‐fat dairy (yes)	1.11 (0.83–1.47)	1.09 (0.82–1.00)	0.90 (0.56–1.45)	0.83 (0.57–1.20)	0.85 (0.58–1.24)	0.57 (0.21–1.45)
High‐fat meat (yes)	1.36 (1.01–1.84)	1.38 (1.02–1.86)	**1.99 (1.12–3.52)**	1.05 (0.73–1.51)	1.08 (0.75–1.56)	0.77 (0.25–2.37)
Fried food (yes)	0.74 (0.57–0.96)	0.72 (0.55–0.95)	0.67 (0.43–1.06)	0.79 (0.57–1.09)	0.90 (0.64–1.25)	1.71 (0.76–3.86)
Consuming mayonnaise (yes)	1.04 (0.76–1.41)	1.02 (0.75–1.40)	0.93 (0.57–1.50)	0.84 (0.59–1.19)	0.95 (0.66–1.36)	1.32 (0.47–3.71)
Using special measuring cup for adding oil (no)	1.32 (1.01–1.72)	1.36 (1.03–1.78)	1.20 (0.74–1.96)	1.01 (0.74–1.37)	1.00 (0.73–1.35)	0.44 (0.16–1.23)
Fast food (yes)	1.05 (0.76–1.45)	1.03 (0.72–1.49)	1.40 (0.71–2.78)	0.93 (0.61–1.41)	1.34 (0.82–2.22)	**3.76 (1.09–13.0)**
Eating salad (no)	1.65 (1.22–2.22)	1.68 (1.24–2.27)	**2.95 (1.75–4.99)**	1.15 (0.82–1.60)	1.03 (0.74–1.45)	1.47 (0.48–4.48)
Boiled food (no)	0.83 (0.64–1.07)	0.82 (0.62–1.07)	0.76 (0.48–1.19)	0.78 (0.57–1.08)	0.89 (0.64–1.24)	1.71 (0.76–3.86)

*Note:* Results are expressed as hazard ratio and (95% confidence interval). Model I: Adjusted for age; Model II: Adjusted for age, smoking, economic status, physical activity and educational level. Significant (*p* < 0.05) results are indicated in bold.

### 3.3 Association Between DHS and Dyslipidemia Risk

The baseline participants' characteristics based on the quartiles of the DHS are presented in Table 4. The range of the DHS was 9.59 to 12.2 for males, 10.87–12.52 for females, and 9.61 to 11.53 for the total population. Significant associations were observed between the quartiles of DHS and factors such as age, sex, weight, BMI, SBP and DBP, physical activity, and educational level (*p* < 0.05). Table 5 presents the dyslipidemia data according to the DHS for males, females, and the total population. The results indicate that males with the highest DHS (quartile 4) had a 3.56 times higher risk (95% CI: 1.95–6.51, *p* trend = 0.001) of developing dyslipidemia compared with those in the lowest quartile (quartile 1) after adjusting for all potential confounders. This association was not observed in females but p for trend was significant (*p* trend < 0.05). Additionally, in the total population, after adjusting for confounders, the risk of dyslipidemia (HR = 2.58; 95% CI: 1.50–4.43) was significantly higher in individuals with the highest DHS than in those in the lowest quartile. A linear trend was noted between DHS and the risk of dyslipidemia in both males and the total population (*p* trend = 0.001).

**TABLE 4:** Baseline participants' characteristics based on quartiles of dietary habits score.

Variable	Dietary habits score				*p* value
	Q1 Median: 9.61 (*n* = 162)	Q2 Median: 10 (*n* = 142)	Q3 Median: 10.56 (*n* = 147)	Q4 Median: 11.53 (*n* = 145)	
Age (year)	38.87 ± 14.03	44.12 ± 13.18	43.18 ± 14.65	48.67 ± 14.61	0.001
Sex (%)					0.008
Male	105 (64.8%)	71 (50.0%)	73 (49.7%)	69 (47.6%)	
Female	57 (35.2%)	71 (50.0%)	74 (50.3%)	76 (52.4%)	
Weight (Kg)	70.64 ± 13.19	71.83 ± 12.00	68.83 ± 13.65	70.65 ± 11.32	0.245
Body mass index (Kg/m^2^)	24.84 ± 4.08	26.46 ± 4.70	25.20 ± 4.29	25.98 ± 4.21	0.005
Waist circumference (cm)	90.11 ± 12.44	92.39 ± 11.96	90.12 ± 12.72	93.05 ± 11.59	0.073
Systolic blood pressure (mmHg)	122.80 ± 13.32	125.37 ± 14.35	121.49 ± 12.35	127.98 ± 17.14	0.001
Diastolic blood pressure (mmHg)	80.58 ± 7.47	81.28 ± 7.32	79.40 ± 7.55	82.30 ± 9.70	0.018
Fasting blood sugar (mg/dL)	91.92 ± 31.29	95.00 ± 32.40	93.01 ± 37.65	99.63 ± 34.43	0.213
Triglyceride (mg/dL)	111.10 ± 41.01	119.99 ± 38.48	120.77 ± 44.11	119.76 ± 40.67	0.124
Cholesterol (mg/dL)	175.40 ± 31.38	177.63 ± 30.59	177.48 ± 33.14	182.15 ± 32.39	0.311
LDL‐cholesterol (mg/dL)	126.32 ± 29.5	121.76 ± 32.5	123.23 ± 32.41	125.92 ± 39.43	0.681
HDL‐cholesterol (mg/dL)	56.84 ± 10.75	57.11 ± 11.29	56.89 ± 11.37	57.54 ± 11.47	0.947
Current smokers (%)					0.59
Yes	27 (16.7%)	27 (19%)	23 (15.6%)	19 (13.1%)	
No	135 (83.3%)	115 (81.0%)	124 (84.4%)	126 (86.9%)	
Physical activity (%)					0.029
Low	56 (49.1%)	59 (68.6%)	52 (59.8%)	64 (69.6%)	
Moderate	46 (40.4%)	24 (27.9%)	30 (34.5%)	25 (27.2%)	
Vigorous	12 (10.5%)	3 (3.5%)	5 (5.7%)	3 (3.3%)	
Economic Status (%)					0.055
Low	20 (22.0%)	8 (11.6%)	23 (34.3%)	15 (31.3%)	
Moderate	39 (42.9%)	33 (47.8%)	23 (34.3%)	21 (43.8%)	
High	32 (35.2%)	28 (40.6%)	21 (31.3%)	12 (25.0%)	
Education (%)					0.001
Primary	53 (35.1%)	66 (47.8%)	68 (48.2%)	91 (63.6%)	
High school	71 (47.0%)	60 (43.5%)	55 (39.0%)	41 (28.7%)	
Academic	27 (17.9%)	12 (8.7%)	18 (12.8%)	11 (7.7%)	

*Note:* Values are mean ± standard deviation, otherwise indicated.

**TABLE 5:** Risk of dyslipidemia according to dietary habits score (DHS) between male and female and total population.

Gender	Dietary habits score				*p* for trend
	Q1	Q2	Q3	Q4	
**Male**	**Median: 9.59** *n* = 105	**Median: 10.21** *n* = 71	**Median: 10.95** *n* = 73	**Median: 12.2** *n* = 69	
Crude	**Reference**	1.43 (1–2.06)	1.96 (1.36–2.83)	2.05 (1.44–2.91)	**0.001**
*p* value		0.051	0.001	0.001	
Model I	**Reference**	1.44 (1–2.08)	**1.97 (1.36–2.83)**	2.06 (1.45–2.95)	**0.001**
*p* value		0.049	**0.001**	0.001	
Model II	**Reference**	1.7 (0.93–3.11)	2.01 (1.1–3.7)	**3.56 (1.95–6.51)**	0.001
*p* value		0.085	0.024	0.001	
**Female**	**Median: 10.87** *n* = 57	**Median: 11.15** *n* = 71	**Median: 11.81** *n* = 74	**Median: 12.52** *n* = 76	
Crude	Reference	0.97 (0.63–1.48)	1.29 (0.84–1.98)	0.9 (0.57–1.42)	1
*p* value		0.88	0.237	0.65	
Model I	Reference	0.98 (0.64–1.5)	1.25 (0.82–1.92)	0.99 (0.63–1.57)	0.7
*p* value		0.92	0.3	0.98	
Model II	Reference	1.15 (0.39–3.37)	3.19 (0.94–10.83)	2.73 (0.86–8.69)	0.034
*p* value		0.795	0.063	0.089	
**Total**	**Median: 9.61** *n* = 162	**Median: 10** *n* = 142	**Median: 10.56** *n* = 147	**Median: 11.53** *n* = 145	
Crude	Reference	1.32 (1.01–1.73)	1.41 (1.08–1.84)	1.71 (1.31–2.23)	0.001
*p* value		0.042	0.011	0.001	
Model I	Reference	1.35 (1.02–1.78)	1.44 (1.1–1.89)	1.72 (1.3–2.3)	0.001
*p* value		0.034	0.008	0.001	
Model II	Reference	1.39 (0.83–2.35)	1.61 (0.98–2.66)	2.58 (1.5–4.43)	0.001
*p* value		0.212	0.06	0.001	

*Note:* Values are mean ± standard deviation, otherwise indicated. Results are reported as hazard ratio and 95% confidence interval. Model I: Adjusted for age; Model II: Adjusted for age, sex, smoking, economic status, physical activity and educational level. To analyze data for total population, the variable of sex was adjusted in both models.

## 4 Discussion

This study found a significant association between dietary habits and the development of dyslipidemia in adults, which supports previous research (Gomez‐Delgado et al. 2021; Nouri, Eskandarzadeh, et al. 2023). The risk of dyslipidemia decreased in participants who engaged in a weight control program after a 9.9‐year follow‐up. This finding aligns with the results of a large systematic review and meta‐analysis of randomized controlled trials, which demonstrated that behavioral weight management programs lead to sustained improvements in cardiometabolic risk factors – including lipid profiles, blood pressure, and glycemic control – for at least 5 years after the program ends, despite partial weight regain (Hartmann‐Boyce et al. 2023). A study has shown that weight loss can significantly decrease TG concentrations (Ayisi Addo et al. 2019). For every one kg of weight lost, TGs are reduced by 1.25 mg/dL, LDL‐C is reduced by 1.67 mg/dL, and HDL‐C increases by 0.37 mg/dL (Hasan et al. 2020). In addition, another study emphasized that weight control strategies are a fundamental and cost‐effective approach to managing hyperlipidemia and reducing cardiovascular risk (Virani et al. 2023).

According to the findings, participants who avoided eating salad and consumed fast food had a higher risk of dyslipidemia. Along with this finding, several studies have suggested that adherence to a diet rich in vegetables and fruits appears to be associated with lower risks for dyslipidemia (Trautwein and Mckay 2020). Studies have shown that dietary fiber, which is abundant in vegetables and fruits, plays an important role in improving lipid profiles and reducing cardiometabolic risk (Ramezani et al. 2024). Phytosterols reduce cholesterol absorption by competing with it and influencing specific genes, while soluble fiber decreases cholesterol levels by binding to it in the digestive system (Brauner et al. 2012; Cabral and Klein 2017; Ghavami et al. 2023). In addition, other compounds reduce the risk of dyslipidemia by reducing oxidative stress and inflammation, improving lipid metabolism, and supporting weight management through possible ways (Surampudi et al. 2016). Considering the significance of consuming vegetables and fruits in decreasing the occurrence of diseases, along with the recommendations from the World Health Organization (WHO), it is crucial to consume at least 400 g, or five portions, of fruits and vegetables daily (World Health Organization 2023). The current study demonstrated that fast food plays a significant role in causing dyslipidemia. Its high levels of unhealthy fats, carbohydrates, sodium, and calories can lead to unfavorable lipid profiles (Bahadoran et al. 2016; Elkhateeb and Alshammary 2017). In addition, Bahadoran et al. stated that regular consumption of fast food is linked to weight gain, increased abdominal fat, insulin resistance, disrupted glucose metabolism, and imbalances in lipid levels. It also contributes to systemic inflammation and oxidative stress (Bahadoran et al. 2016). As a result, the risk of developing type 2 diabetes, metabolic syndrome, and CVD is likely to increase (Bahadoran et al. 2016). While most studies support our findings, some have not observed this association (Bahadoran et al. 2016; Jafri et al. 2021; Sobhani et al. 2021).

Retaining the skin on poultry has been associated with a reduced risk of dyslipidemia, likely due to its content of unsaturated fats. The fat profile of chicken skin, where unsaturated fats predominate over saturated fats (65% vs. 34%), can contribute to improved lipid profiles (Soliman and El‐Shattory 2020). In contrast to previous research, our findings indicated that disregarding food label information may be associated with a reduced risk of dyslipidemia (Kim et al. 2015). Research indicates that while attention to food labels may have reduced total fat intake, it has not significantly altered the consumption of salt, carbohydrates, saturated fatty acids, whole grains or protein (Shangguan et al. 2019). In general, this could be attributed to the simplicity of the labels, a general lack of public awareness, and the tendency of individuals to focus primarily on fat content while overlooking other important factors. Some dietary habits, like not using a measuring cup for salt, are associated with increased dyslipidemia risk. However, this association disappears when potential confounding variables are adjusted, suggesting that some variables may significantly influence the association between dietary habits and the risk of dyslipidemia. Other habits show no correlation with dyslipidemia, in line with several studies.

Since individuals often consume a combination of foods at each meal – such as fried foods and fast food alongside salad and mayonnaise –it is essential to evaluate an overall score for eating habits. This score can assess the cumulative impact of these choices. Previous research has utilized this scoring system to examine dietary habits and lifestyles in relation to the occurrence of diseases, blood sugar levels, and lipid profiles. Results from these studies have indicated that DHS or dietary patterns can serve as better predictors for conditions such as hyperglycemia, hypertension, dyslipidemia, and chronic disease (Sarebanhassanabadi et al. 2020; Esmaillzadeh et al. 2007). In this study, DHS could provide more realistic risk estimates for the association between overall dietary habits and dyslipidemia. In addition, we considered some effective confounders that might affect the association between DHS and the risk of dyslipidemia to obtain valuable and consistent results. After adjusting for key confounders, analysis over approximately 10 years revealed that participants in the highest DHS quartile were 2.58 times more likely to develop dyslipidemia than those in the lowest quartile. This aligns with broader evidence, including studies on the Dietary Quality Index‐International (DQI‐I) and the Healthy Eating Index‐2015 (HEI‐2015), which similarly link higher diet quality scores to favorable lipid profiles (Nouri, Gerami, et al. 2023). Moreover, an analysis of the same cohort revealed that individuals in the highest quintile of the DHS are three times more likely to develop low HDL‐C than those in the lowest quintile (Sarebanhassanabadi et al. 2020). These findings support that DHS offers a broader assessment of food and nutrient consumption and may be more indicative of dyslipidemia risk than analyses of individual foods or nutrients (Toft et al. 2007; Nouri, Gerami, et al. 2023; Sarebanhassanabadi et al. 2020).

In addition, sex‐stratified analyses revealed sex differences in the association between DHS and dyslipidemia risk. Males with higher DHS had a 3.56‐fold increased risk of developing dyslipidemia compared with those with lower scores. The analysis revealed a linear association between DHS and the risk of developing dyslipidemia in male subjects and the total population. This association was not observed in females, which may be explained by sex‐specific hormonal factors, fat distribution, and dietary habits and lifestyle choices (Goossens 2017; Mo et al. 2025). These factors lead to a lower impact of unhealthy diets on lipid profiles in females, especially before menopause (Mo et al. 2025). Analysis revealed no significant quartile‐specific associations between dietary patterns and dyslipidemia risk in females but showed a significant trend (*p* trend < 0.05) indicating a graded relationship that requires further investigation. In males, lack of weight control, nonconsumption of salad, and high‐fat meat intake emerged as independent dyslipidemia risk factors, whereas fast food consumption elevated risk in females—patterns aligning with sex‐specific preferences, such as males favoring larger portions of red meat over females (Rosenfeld and Tomiyama 2021). Enani et al. reported higher dyslipidemia risk in females with medium‐to‐high fruit/vegetable intake, potentially due to calorie‐dense salad dressings (Enani et al. 2020). Collectively, these findings underscore the need to consider biological (hormonal, adiposity) and behavioral (food choices, ready‐to‐eat foods) mechanisms in interpreting diet–dyslipidemia association, advocating for sex‐specific strategies in nutritional epidemiology and cardiovascular prevention.

This study has several strengths; this study was the first survey to investigate the association between DHS and 9.9‐year incidence of dyslipidemia in urban adults' population in central Iran. By developing this scoring method, we can study the association between overall dietary habits and dyslipidemia, rather than just one dietary habit. When interpreting our results, it is important to consider a few limitations. In the study, 33.5% of participants without dyslipidemia during repeated measurements were lost to follow‐up. Despite adjusting for key confounders, the possibility of residual confounding and attrition bias cannot be fully ruled out, as participants lost to follow‐up differed from those who remained in terms of age, sex, economic status and educational levels. Also, lifestyle factors may have changed over the follow‐up period, possibly reducing the study's power to accurately estimate risk. Furthermore, the wide confidence intervals for some estimates, particularly in sex‐stratified analyses, suggest a need for caution in interpretation and warrant replication in larger samples. While advanced machine learning techniques like Random Forest could offer complementary insights into predictor importance in future studies, our use of Cox regression was chosen to provide directly interpretable measures of association (HR) for clinical and public health application.

### 4.1 Policy Implications and Recommendations

The findings of this study carry direct implications for public health strategy and clinical practice. Public health programs, particularly in urban settings, should prioritize nutritional education focused on weight management, increased vegetable intake, and reduced consumption of fast food. Furthermore, the DHS emerges as a potentially valuable, simple tool for the early identification of individuals with unhealthy dietary habits in primary care and community health screenings.

## 5 Conclusion

In conclusion, this study demonstrates that ineffective weight management, low salad consumption, and frequent fast food intake are significantly associated with an increased risk of dyslipidemia. Additionally, a positive significant association was found between the highest quartile of DHS and the 9.9‐year incidence of dyslipidemia in an urban adult population. However, these findings should be interpreted with caution when generalizing to a broader population. From a practical standpoint, the DHS could serve as a simple, valuable tool for early identification of high‐risk individuals in both clinical and community health settings. Integrating such a score into routine health screenings may facilitate timely, targeted dietary counseling and behavioral interventions. These results point to specific public health priorities: nutrition programs should emphasize weight management, increased vegetable intake, and reduced fast food consumption, particularly in urban settings. Implementing these targeted, evidence‐based dietary strategies can support primary prevention efforts, helping to lower the incidence of dyslipidemia and subsequent cardiovascular risk.

## Author Contributions

**Mohammadtaghi Sarebanhassanabadi:** conceptualization, methodology, software, supervision, validation, resources, writing – original draft, data curation, project administration, investigation, formal analysis. **Abolfazl Mohammadi:** conceptualization, investigation, writing – original draft. **Pedro Marques‐Vidal:** writing – review and editing. **Seyedmostafa Seyedhosseini:** writing – original draft. **Seyedeh Mahdieh Namayandeh:** writing – review and editing. **Hanieh Montazeri Hedesh:** writing – original draft. **Sima Mozafari:** conceptualization, writing – original draft, supervision, investigation.

## Funding

This study received no financial support or external funding from public, commercial, or nonprofit sources.

## Ethics Statement

Ethical approval was granted by the Ethics Committee at Shahid Sadoughi University of Medical Sciences (Ethics Code: IR.SSU.MEDICINE.REC.1395.287). All procedures conformed to the Declaration of Helsinki.

## Consent

Informed consent was obtained from all participants for the use of their data in analyses conducted during both the initial and follow‐up phases.

## Conflicts of Interest

The authors declare no conflicts of interest.

## Data Availability

The data that support the findings of this study are available on request from the corresponding author. The data are not publicly available due to privacy or ethical restrictions.
